# Multi-photon excitation microscopy

**DOI:** 10.1186/1475-925X-5-36

**Published:** 2006-06-06

**Authors:** Alberto Diaspro, Paolo Bianchini, Giuseppe Vicidomini, Mario Faretta, Paola Ramoino, Cesare Usai

**Affiliations:** 1 LAMBS-MicroScoBio Research Center, Department of Physics, University of Genoa, Via Dodecaneso 33, 16146 Genova, Italy; 2 IFOM The FIRC Institute for Molecular Oncology Foundation, Via Adamello, 16, 20139 Milan, Italy; 3 IFOM-IEO Consortium for Oncogenomics European Institute of Oncology, via Ripamonti 435, 20141 Milan, Italy; 4 DIPTERIS – Department for the Study of the Territory and its Resources, University of Genoa, Corso Europa 26, 16132 Genova, Italy; 5 CNR- National Research Council, Institute of Biophysics, Via De Marini, 6, 16149 Genova, Italy

## Abstract

Multi-photon excitation (MPE) microscopy plays a growing role among microscopical techniques utilized for studying biological matter. In conjunction with confocal microscopy it can be considered the imaging workhorse of life science laboratories. Its roots can be found in a fundamental work written by Maria Goeppert Mayer more than 70 years ago. Nowadays, 2PE and MPE microscopes are expected to increase their impact in areas such biotechnology, neurobiology, embryology, tissue engineering, materials science where imaging can be coupled to the possibility of using the microscopes in an active way, too. As well, 2PE implementations in noninvasive optical bioscopy or laser-based treatments point out to the relevance in clinical applications. Here we report about some basic aspects related to the phenomenon, implications in three-dimensional imaging microscopy, practical aspects related to design and realization of MPE microscopes, and we only give a list of potential applications and variations on the theme in order to offer a starting point for advancing new applications and developments.

## 1. Introduction

There have been a variety of reasons for the continuing growth of interest in optical microscopy in spite of the low resolution with respect to modern scanning probe or electron microscopy [1]. The main reason lies in the fact that optical microscopy is still considered unique in allowing the 4D (x-y-z-t) examination of biological systems in a hydrated state in living samples or under experimental conditions that are very close to living or physiological states. This evidence coupled to fluorescence labelling and other advances in molecular biology permits to attack in an effective way the complex and delicate problem of the connection between structure and function in biological systems [2-6]. Within this framework, inventions in microscopy were stimulated, and contributed to the evolution of the optical microscope in its modern forms [7,8]. Multiphoton excitation microscopy is an important part of this progress in the field of microscopy applied to the study of biological matter from the inventions of the confocal microscope [9] and of the atomic force microscope [10]. Nowadays, confocal and multiphoton microscopes can be considered the imaging workhorses of life science laboratories [11].

Multiphoton excitation microscopy (MPE) has its roots in two-photon excitation (2PE) microscopy whose story dates back more than 70 years. In 1931, Maria Goeppert-Mayer published her brilliant doctoral dissertation on the theory of two-photon quantum transitions in atoms and established the theoretical basis behind 2PE [12]. This photophysical effect was experienced after the development of laser sources, as well, other non-linear related effects were also observed in the 60s and 70s [13,14]. In 1976, Berns reported about a probable two-photons effect as a result of focusing an intense pulsed laser beam onto chromosomes of living cells [15], and such interactions form the basis of modern nanosurgery [16] and targeted transfection [17]. One has to wait until 1978 to find the description of the first nonlinear scanning optical microscope with depth resolution. The reported microscopic imaging was based on second-harmonic generation (SHG) and the possibility of performing 2PE microscopy was outlined [18]. Unfortunately, applications in biology were hampered due by the high peak intensities required for priming 2PE fluorescence. This obstacle was overcome with the advent of ultrashort and fast pulsed lasers in the 80s [19]. Denk and colleagues in a seminal paper on 2PE laser scanning fluorescence microscopy clearly demonstrated the capability of 2PE microscopy for biology [20]. This fact brought a "new deal" in fluorescence microscopy [21-23]. Such a leap in scientific technology stimulated disparate disciplines and several variations on the theme extending studies from the tracking of individual molecules within living cells to the observation of whole organisms [24-28].

### 2. Foundations of 2PE microscopy

**Figure 1:**
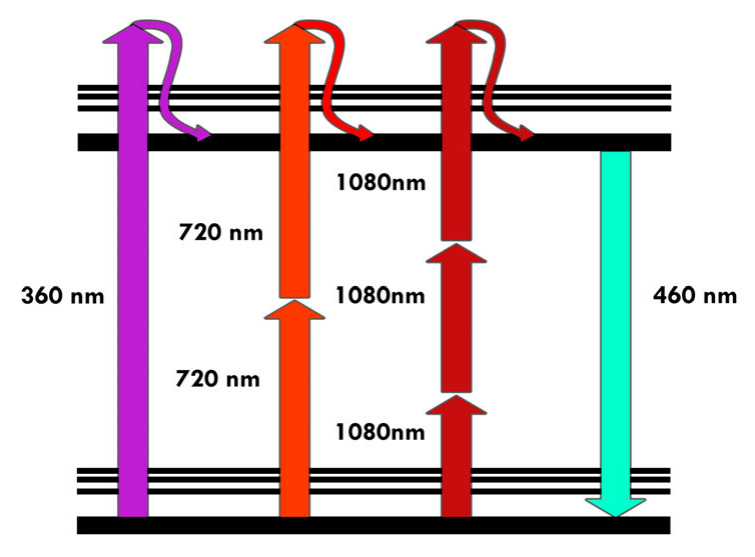
**Perrin-Jablonski fluorescence diagram. **Simplified Perrin-Jablonski scheme for 1PE, 2PE and 3PE. Once the excited state has been reached the subsequent fluorescent emission is the very same for the three different modalities of excitation.

Two-photon excitation of fluorescent molecules is a non-linear process related to the simultaneous absorption of two photons whose total energy equals the energy required for the more familiar one-photon excitation (1PE) [29]. The excitation process of a fluorescent molecule under 1PE typically requires photons in the ultraviolet or blue/green spectral range. Under sufficiently intense illumination, usually provided by a laser source, the very same process, i.e. excitation of a fluorescent molecule from the ground to the excited state, can take place in the infrared spectral range. This situation is illustrated in figure 1 using a Perrin-Jablonski diagram: the sum of the energies of the "two" infrared photons colliding with the very same molecule has to be greater than the energy gap between the molecule's ground and excited states. Since 2PE excitation requires at least two statistically "independent" photons for each excitation process, its rate depends on the square power of the instantaneous intensity. 3PE and higher photon excitation is also possible. This implies that deep ultraviolet (UV) microscopy can be performed without having the disadvantages related to UV-matter interactions, i.e. polymerization or temperature effects. However, our treatment will be mainly conducted in terms of 2PE for sake of simplicity but any step can be extended to MPE.

The most popular relationship about 2PE is related to the practical situation of a train of beam pulses focused through a high numerical aperture (NA) objective, with a duration τ_p _and f_p_repetition rate. The advantage of using a train of repeated beam pulses is given by the fact that high peak power can be used for priming the process resulting on an average power tolerated by the biological system under investigation. Under controlled conditions, the probability, n_a_, that a certain fluorophore simultaneously absorbs two photons during a single pulse, in the paraxial approximation is given by [20]



where P_ave _is the average power of the illumination beam, δ_2 _is the two-photon cross section of the fluorescent molecule, and λ is the excitation wavelength. Introducing 1 GM (Goppert-Mayer) = 10^-58 ^[m^4 ^s], for a δ_2 _of approximately 10 GM, focusing through an objective of NA >1, an average incident laser power of ≈ 1–50 mW, operating at a wavelength ranging from 680 to 1100 nm with 80–150 fs pulsewidth and 80–100 MHz repetition rate, one should get fluorescence without saturation. It is convenient that for optimal fluorescence generation, the desirable repetition time of pulses should be on the order of typical excited-state lifetime, which is a few nanoseconds for commonly used fluorescent molecules. For this reason the typical repetition rate is around 100 MHz, i.e. one order of magnitude slower than typical fluorescence lifetime. As well, during the pulse time (10^-13 ^s of duration and a typical lifetime in the 10^-9 ^s range) the molecule has insufficient time to relax to the ground state. This can be considered a prerequisite for absorption of another photon pair. Therefore, whenever n_a _approaches unity saturation effects start occurring. Such a consideration allows one to optimize optical and laser parameters in order to maximize the excitation efficiency without saturation. In case of saturation the resolution is declining and worsening the image. It is also evident that the optical parameter for enhancing the process in the focal plane is the lens numerical aperture, NA, even if the total fluorescence emitted is independent from this parameter. This value is usually kept around 1.3–1.4. As example, for fluorescein that possesses a δ_2 _≅38 GM at 780 nm, using NA = 1.4, a repetition rate of 100 MHz and pulse width of 100 fs within a range of P_ave _assumed 1, 10, 20 and 50 mW, from equation (1) one has n_a_≈ 5930 (P_ave_)^2^. This means that, as function of the average excitation power 1, 10, 20, 50 mw one gets 5.93 10^-3^, 5.93 10^-1^, 1.86, 2.965 respectively, with saturation starting at 10 mW. The related rate of photon emission per molecule, at a non saturation excitation level, in absence of photobleaching, is given by n_a _multiplied by the repetition rate of the pulses, i.e. approximately 5·10^7 ^photons s^-1^. It is worth noting that when considering the effective fluorescence emission one should consider a further factor given by the so-called quantum efficiency of the fluorescent molecules. It is worth noting that the fluorophore emission spectrum results independent of the excitation mode from 1PE to MPE like the quantum efficiency.

Now, even if the quantum-mechanical selection rules for MPE differ from those for one-photon excitation, several common fluorescent molecules can be used. Unfortunately, the knowledge of 1PE cross-section for a specific fluorescent molecule does not allow any quantitative prediction of the two-photon trend. The only "rule of thumb" that one can use states that a 2PE cross-section peak can be expected at a 2 folds wavelength with respect to the 1PE case. Table 1 summarises the excitation properties of some popular fluorescent molecules under 2PE regime.

**Table 1:** 2PE excitation parameters.

FLUOROPHORES	**λ (nm)**	**ηδ_2_**	**δ_2_**
**Extrinsic fluorophores**			
Bis-MSB	691/700	6.0 ± 1.8	6.3 ± 1.8
Bodipy	920	17 ± 4.9	-
Calcium Green	740–990	-	~80
Calcofluor	780/820	-	-
Cascade blue	750–800	2.1 ± 0.6	~3
Coumarin 307	776, 700–800	19 ± 5.5	~20
CY2	780/800	-	-
CY3	780	-	-
CY5	780/820	-	-
DAPI (free)	700/720	0.16 ± 0.05	~3.5 *
Dansyl	700	1	-
Dansyl Hydrazine	700	0.72 ± 0.2	-
Dil	700	95 ± 28	-
Filipin	720	-	-
FITC	740–820	-	~25–38 *
Fluorescein (pH ~11)	780	-	38 ± 9.7
Fura-2 (free)	700	11	-
Fura-2 (high Ca)	700	12	-
Hoechst	780/820	-	-
Indo-1 (free)	700	4.5 ± 1.3	12 ± 4
Indo-1 (high Ca)	590/700	1.2 ± 0.4	2.1 ± 0.6
Lucifer Yellow	840–860	0.95 ± 0.3	~2
Nile Red	810	-	-
Oregon Green Bapta 1	800	-	-
Rhodamine B	840	-	210 ± 55
Rhodamine 123	780–860	-	-
Syto 13	810	-	-
Texas red	780	-	-
Triple probe (Dapi, FITC, and Rhodamine)	720/740	-	-
TRITC (rhodamine)	800–840	-	-

## 3. Optical implications of 2PE

### 3.1 Optical sectioning and confocal imaging

**Figure 2:**
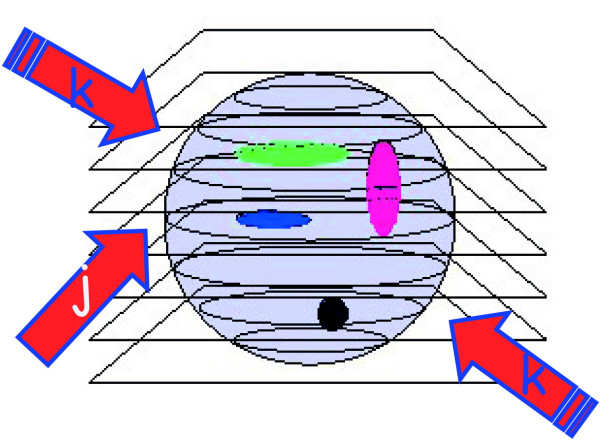
**Optical sectioning scheme. **A three-dimensional sample can be sketched as a series of optical slices. Let's call slice j the one containing the geometrical focus of the objective and refer to the adjacent planes as k slices. The sample contains a three-dimensional distribution of fluorescently labelled molecules whose intensity distribution is I, slice by slice. The thickness of each optical slice is approximately one half of the axial resolution, say ≈λ/2.

The possibility of the three-dimensional reconstruction of the volume distribution of intensive parameters, as fluorescence emission, from biological systems is one of the most powerful properties of the optical microscope. To collect optical slices from a three-dimensional object the so-called optical sectioning [3,11] technique is used as depicted in figure 2. It is essentially based on a fine z stepping either of the objective or of the sample stage, coupled with the usual x-y image capturing. The synchronous x-y-z scanning allows the collection of a set of two-dimensional images, which are somehow affected by signal cross talk from other planes from the sample. In fact, the observed image O_j_, obtainable when positioning the geometrical focus of the lens at a certain plane" j "within the specimen, is produced by the true fluorescence distribution I_j _at plane "j", distorted by the microscope in some way that can be described by a function S, plus differently distorted contributions from adjacent "k" planes positioned above and below the actual plane, and noise N. Using a convenient and appropriate formalism one has:

O_j _= I_j_S_j _+ ∑_k≠j _I_k_S_k _+ N.     (2)

Equation (2) reflects the fact that when a set of two-dimensional images is acquired at various focus position and under certain conditions, in principle, one can recover the 3D shape of the object, described by the intensive parameter I, by solving the above set of equations and finding the best estimate for I, slice by slice. By this procedure, unwanted light can be computationally removed combining the image data from a stack of "k" images. Such an operation can be optically performed using some physical stratagems that are behind confocal and MPE/2PE scanning microscopy.

**Figure 3:**
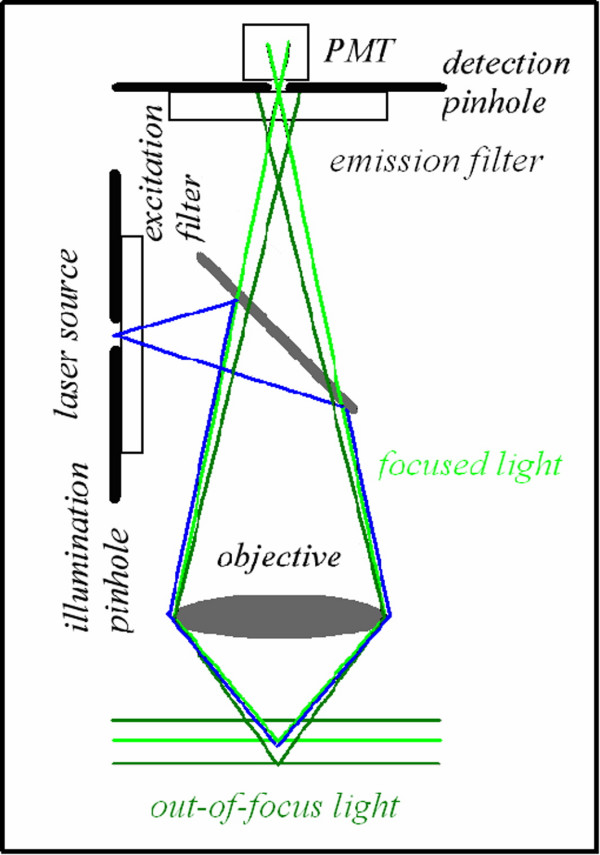
**Confocal optical pathways. **An illumination and a detection pinhole are placed in the optical pathway. The detection pinhole – the mask – is placed in front of the detector at a plane that is conjugate to the in-focus or "j" plane, such that the illumination spot and the pinhole aperture are simultaneously focused at the same specimen volume. This coincidence of the illumination and detected volume is responsible for confocality. The illumination pinhole allows to perform pointlike scanning.

In confocal microscopy, the observed image is built up, scanning the sample point by point, by adding information from x-y-z sampled regions. The price to pay, or the main drawback, is that image formation is not as immediate as widefield techniques in which the whole image is acquired at the same time. This is due to the fact that in confocal microscopy the specimen is sequentially illuminated point by point and at the same time all is masked but the illuminated in-focus regions for providing return light to the detector. As shown in figure 3 an illumination and a detection pinhole are placed in the optical pathway. The detection pinhole – the mask – is placed in front of the detector at a plane that is conjugate to the in-focus or "j" plane, such that the illumination spot and the pinhole aperture are simultaneously focused at the same specimen volume. This coincidence of the illumination and detected volume is responsible for confocality. The final effect of such an optical implementation is that out-of-focus contributions are excluded from the detector surface and the observed image, O_j_, is close to the true distribution of the fluorescence intensity I, plane by plane during the x-y-z scanning operations. This allows performing optical sectioning.

### 3.2 The 2PE optical case

In terms of optical implications the two-photon effect has the important consequence of limiting the excitation region to within a sub-femtoliter volume. This means that the emission region is intrinsically confocal. The resulting 3D confinement in terms of image formation process can be described by means of consolidated optical considerations [30]. Using a certain excitation light at a wavelength λ, the intensity distribution within the focal region of an objective having numerical aperture NA = n sin (α) is given, in the paraxial regime, by



where J_0 _is the 0th order Bessel function, ρ is a radial coordinate in the pupil plane, n is the refractive index of the medium between the lens and the specimen, (α) is the semi-angle of aperture of the lens [31],



and



are dimensionless axial and radial coordinates, respectively, normalized to the wavelength. Now, the intensity of fluorescence distribution within the focal region has a I(u, v) behaviour for the 1PE case [31]. In case of 2PE one has to consider a double wavelength and a square behaviour, i.e. **I**^2^**(u/2, v/2)**. As compared with the 1PE case, the 2PE emission intensity distribution is axially confined.

In fact, considering the integral over ν, keeping *u *constant, its behaviour is constant along z for one-photon and has a half-bell shape for 2PE. This behaviour is responsible of the 3D discrimination property of 2PE, i.e. of the optical sectioning properties of the 2PE microscope.

**Figure 4:**
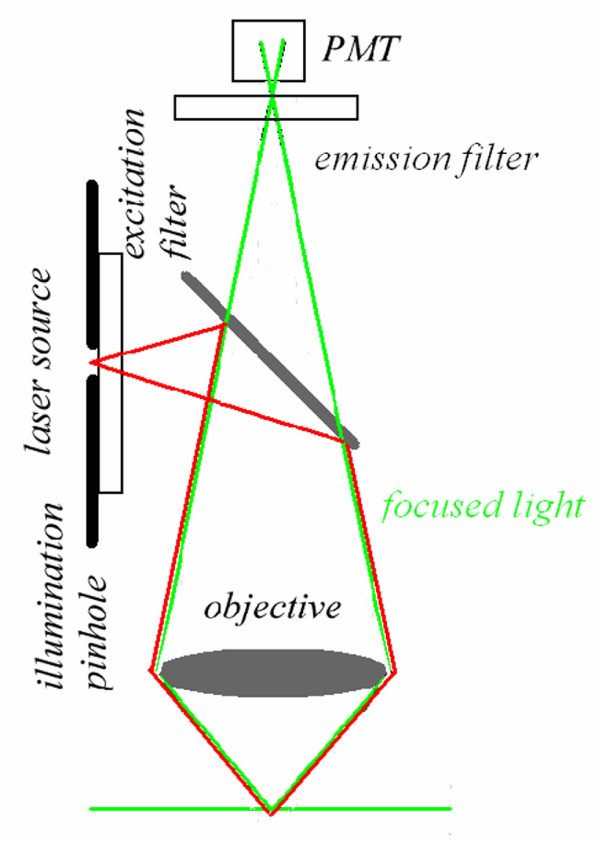
**MPE simplified optical pathways. **In the MPE optical pathways the emission pinhole is removed since the only emitted light reaching the sensor is coming from the currently point scanned volume in the sample. No other fluorescence signal is produced elsewhere.

Now, the most interesting aspect is that the excitation power falls off as the square of the distance from the lens focal point, within the approximation of a conical illumination geometry [31]. In practice this means that the square relationship between the excitation power and the fluorescence intensity brings about the fact that 2PE falls off as the fourth power of distance from the focal point of the objective. This fact implies that those molecules away from the focal region of the objective lens do not contribute to the image formation process and are not affected by photobleaching or phototoxicity. Since these molecules are not involved in the excitation process, a confocal-like effect is obtained without the necessity of a confocal pinhole. It is immediately evident that in this case the optical sectioning effect is obtained in a physically different way with respect to the confocal case. Accordingly the optical set-up is simplified, under some aspects, see figure 4.

**Figure 5:**
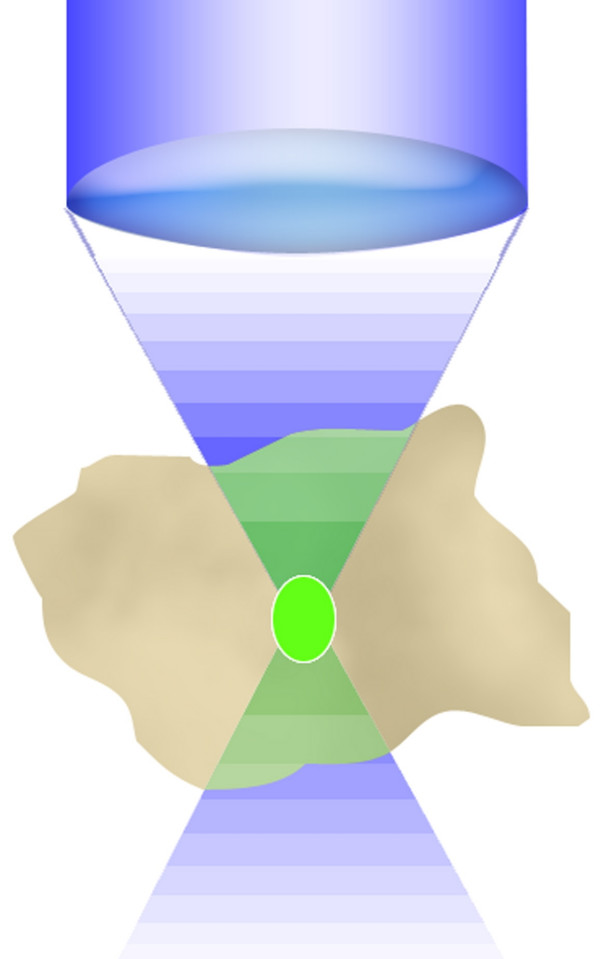
**Confocal fluorescence emission distribution. **The emission process, in green, under blue 1PE excitation is broadened in the whole double cone excitation shape within the analyzed cell.

**Figure 6:**
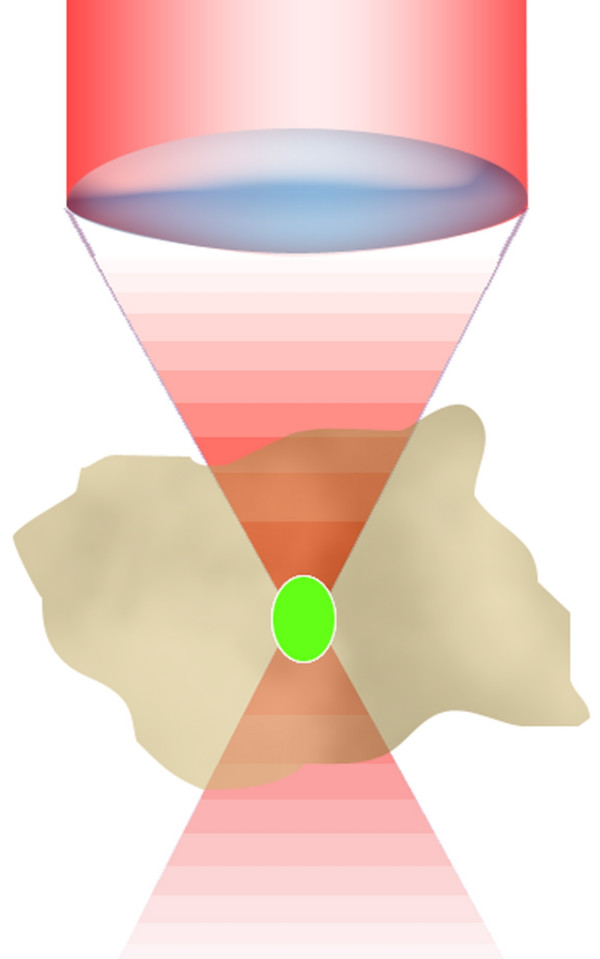
**MPE fluorescence emission distribution. **Confinement of the emission process, in green, under red 2PE. Under 2PE the only molecules excited are those confined in a small subfemtoliter volume at the illumination beam focus position. This is particularly relevant for the photobleaching process that is globally reduced with respect to the 1PE case. The capacity of the volume can be approximatively calculated by using the resolution parameters of the optical system, since they are indicators of the volume containing the maximum photon density. This is valid only for non saturated processes. Under saturation of fluorescence beam intensity plays an important role in the emission shape and optical resolution.

**Figure 7:**
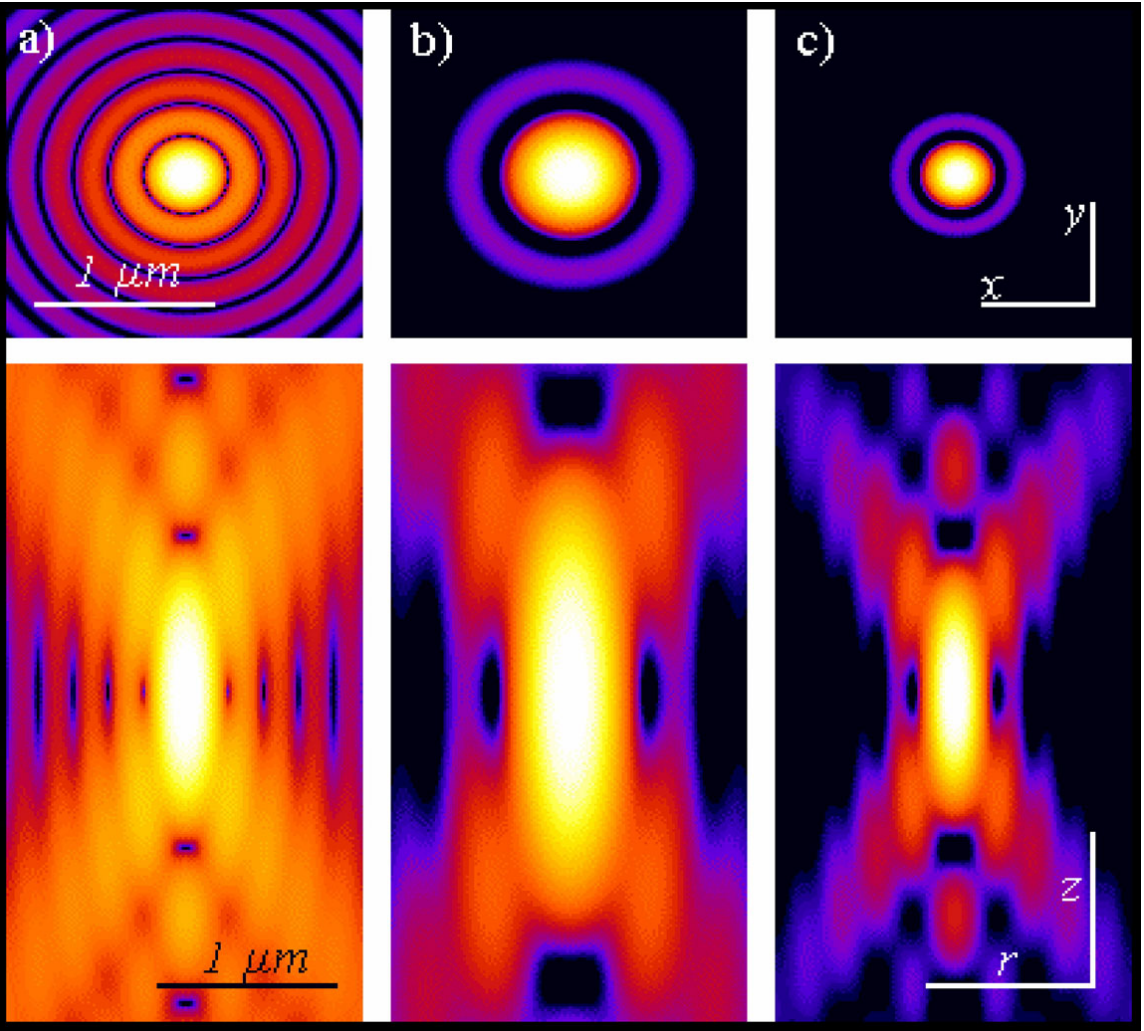
**Pointlike emitter optical response. **From left to right: calculated x-y (above) and r-z (below) intensity distributions, in logarithmic scale, for a point like source imaged by means of wide-field, 2PE and confocal microscopy. Both 2PE and confocal shapes exhibit a better signal to noise ratio than widefield case. 2PE distribution is larger due the fact that a wavelength twice than in the wide-field and confocal case is responsible for the intensity distribution. Such intensity distributions are also known as point spread functions of the related microscopes. Optical conditions: excitation wavelengths are 488 nm and 900 nm for 1PE and 2PE, respectively; emission wavelength is 520 nm; numerical aperture is 1.3 for an oil immersion objective with oil refractive index value set at 1.515.

Figure 5 and figure 6 show the differences in terms of excitation-emission process between confocal and multiphoton schemes, respectively. The consequences of the spatial confinement of the MPE result in a consequent confinement of the emitted fluorescence: in the confocal case all the molecules within the double cone of excitation are involved in the light-matter interaction while in the MPE case such interaction is restricted to a small volume centred at the geometrical focus of the objective. The immediate consequence is that a 2PE microscope is an intrinsically three-dimensional image formation system. This fact has also very important consequences on the photobleaching processes. So far, in the 2PE case no fluorescence has to be removed from the detection pathway since fluorescence can exclusively come from a small focal volume that has a capacity of the order of fraction of femtoliter. In fact, in 2PE over 80% of the total intensity of fluorescence comes from a 700–1000 nm thick region about the focal point for objectives with numerical apertures in the range from 1.2 to 1.4 [30]. The fact that the background signal coming from adjacent planes tends to zero produces a sort of compensation for the reduced spatial resolution due to the utilization of a wavelength that is twice with respect to the 1PE case, as shown in figure 7. On the other hand, the utilisation of infrared wavelengths instead of UV-visible ones allows achieving a deeper penetration than in conventional case [32]. This is due to the fact that the scattering effect is proportional to the inverse fourth power of the wavelength. Thus the longer wavelengths used in 2PE, or in general in MPE, will be scattered less than the ultraviolet-visible wavelengths used for conventional excitation allowing to reach fluorescence targets in depth within thick samples (approx. 1 mm). It has been shown that two-photon fluorescence images can be obtained throughout almost the entire grey matter of the mouse neocortex by using optically amplified femtosecond pulses. The achieved imaging depth approaches the theoretical limit set by excitation of out-of-focus fluorescence [33]. The fluorescence light emitted, on the way back, can be more efficiently collected using a large area detector since it can uniquely come from the sub-femtoliter 2PE volume of event.

## 4. Practical aspects for the realization of a 2PE microscope

**Figure 8:**
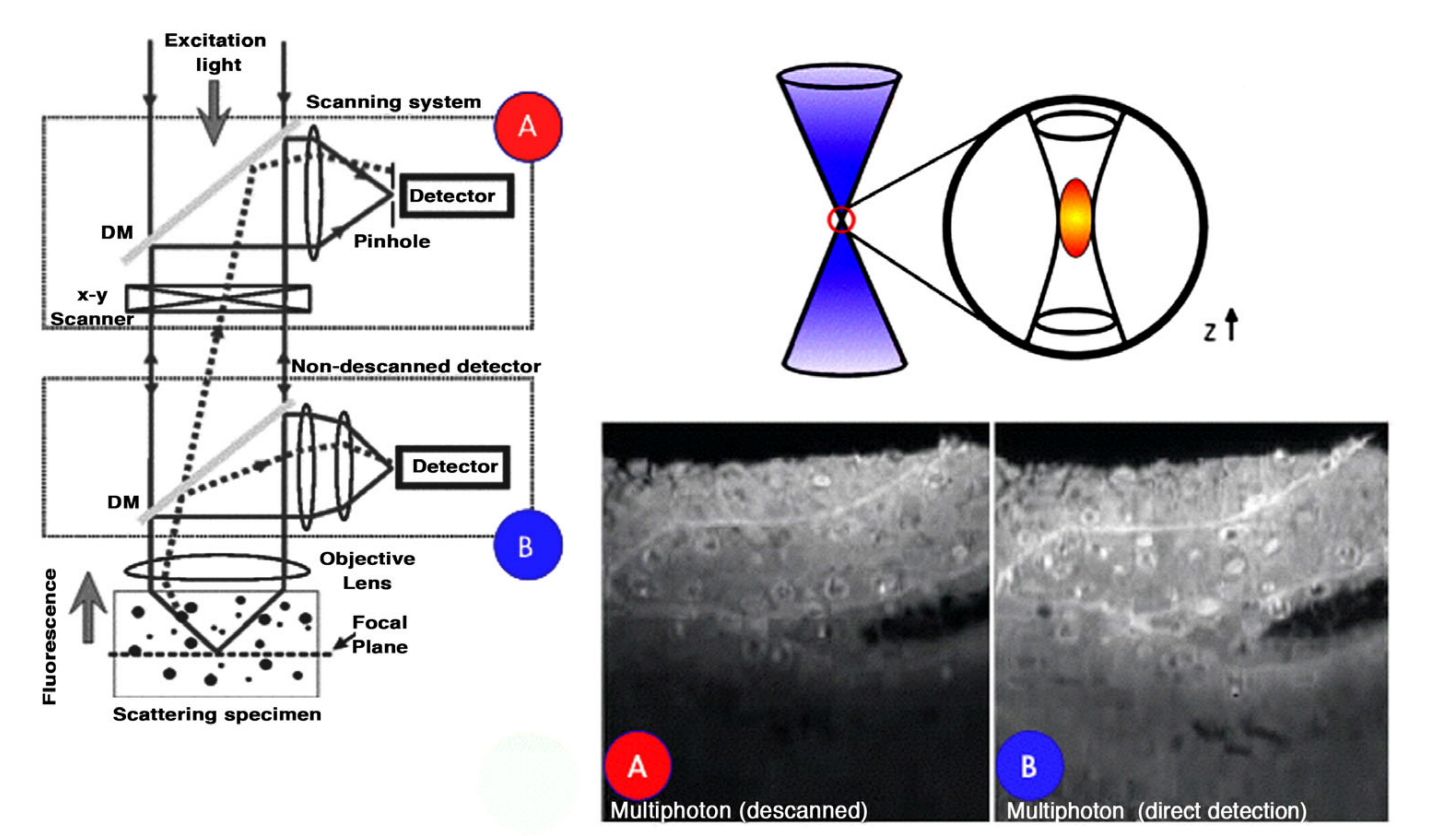
**MPE simplified optical schemes. **Descanned (red dot) and non-descanned (blue dot) schemes for 2PE microscopy. Due to the confinement of the excitation process when operating in non-descanned mode the collection efficiency in depth is increased as shown by the two side views from the very same thick scattering sample (Courtesy of Mark Cannel and Christian Soeller; image inset courtesy of Ammasi Periasamy).

**Figure 9:**
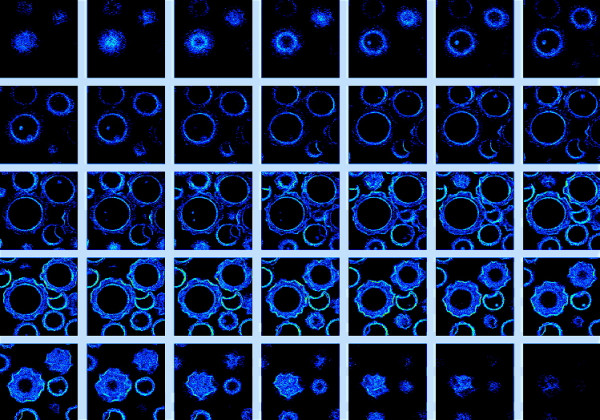
**Optical sectioning using 2PE autofluorescence. **2PE optical sectioning of *Colpoda maupasi *resting cysts, 21–32 μm average dimensions. Encystment is particularly widespread in species living in ephemeral fresh-water puddles and is induced by exhaustion of the food and drying out. The resulting images have been obtained at LAMBS-MicroScoBio exploiting autofluorescence primed using 740 nm excitation. A Chameleon-XR ultrafast Ti-Sapphire laser (Coherent Inc., USA) and a Nikon PCM2000 confocal scanning head have been used [34]. Linear frame dimension is 70 μm, z steps have been performed every 0.5 μm, and not all the optical sections are imaged.

**Figure 10:**
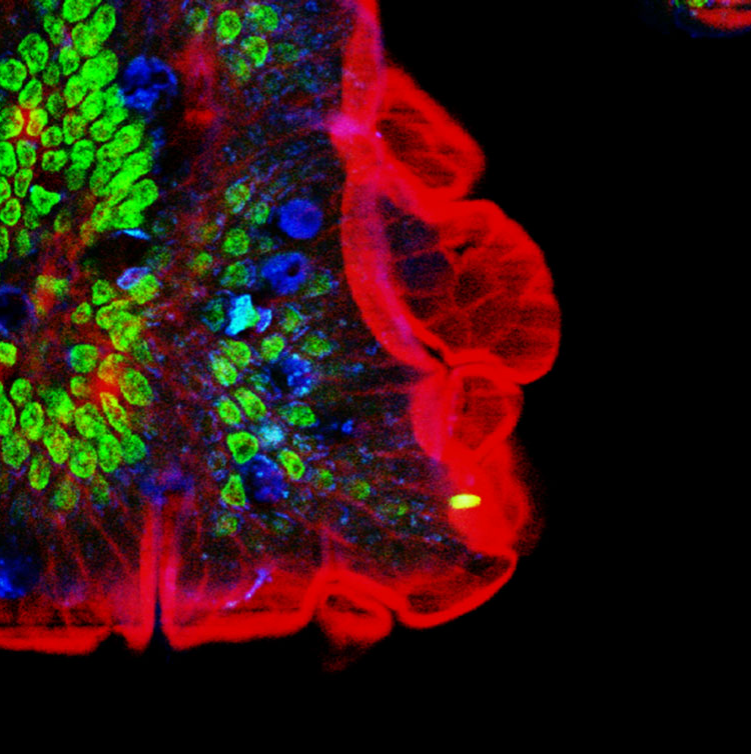
**Multiple fluorescence 2PE imaging. **2PE multiple fluorescence image from a 16 μm cryostat section of mouse intestine stained with a combination of fluorescent stains (F-24631, Molecular Probes). Alexa Fluor 350 wheat germ agglutinin, a blue-fluorescent lectin, was used to stain the mucus of goblet cells. The filamentous actin prevalent in the brush border was stained with red-fluorescent Alexa Flu or 568 phalloidin. Finally, the nuclei were stained with SYTOX ^® ^Green nucleic acid stain. Imaging has been performed at 780 nm, 100 x 1.4 NA Leica objective, using a Chameleon XR ultrafast Ti-Sapphire laser (Coherent Inc., USA) coupled at LAMBS-MicroScoBio with a Spectral Confocal Laser Scanning Microscope, Leica SP2-AOBS.

**Figure 11:**
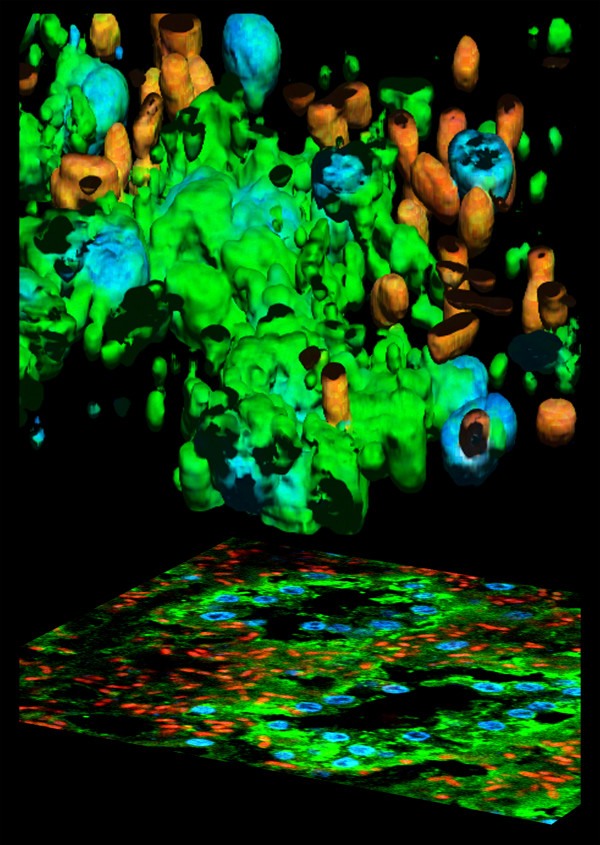
**3D and 2D fluorescence projections. **Pictorial representation of the 3D and 2D projections of multiple fluorescence from a marine sponge, *Chondrilla nucula*. The specimen has been loaded with Alexa 488 fluorescent molecules specific aminobutirric acid (GABA) emitting in green, DAPI for nuclear DNA for the blu component. Red signals are due to the autofluorescence from symbiontic bacteria contamination. Imaging has been perfomed using a Chameleon XR ultrafast Ti-Sapphire laser (Coherent Inc., USA) coupled at LAMBS-MicroScoBio with a Spectral Confocal Laser Scanning Microscope, Leica SP2-AOBS. (Sample availability and preparation, courtesy of Renata Manconi, University of Sassari, Roberto Pronzato and Lorenzo Gallus, University of Genoa).

The main elements to realize a 2PE/MPE architecture, including confocal modality, are the following: high peak-power laser delivering moderate average power (fs or ps pulsed at relatively high repetition rate) emitting infrared or near infrared wavelengths (650–1100 nm), laser sources for confocal 1PE, a laser beam scanning system, high numerical aperture objectives (>1), a high-throughput microscope pathway, a spectral separation module for the emitted signal discrimination, and a high-sensitivity detection system [34]. Figure 8 shows a general scheme for a MPE microscope also illustrating two popular approaches that can be used for image formation, namely: de-scanned and non de-scanned mode. The former uses the very same optical pathway and mechanism employed in confocal laser scanning microscopy. The latter mainly optimises the optical pathway by minimising the number of optical elements encountered on the way from the sample to detectors, and increases the detector area. MPE non-descanned mode allows very good performances providing superior signal-to-noise ratio inside strongly scattering samples [32,33]. In the de-scanned approach pinholes are removed or set to their maximum aperture and the emission signal is captured using the very same optical scanning pathway used for excitation. In the latter, the aim is to optimize the collection efficiency: pinholes are removed and the radiation emitted without passing through the laser beam scanning mirrors. Photomultiplier tubes are the most popular detectors in MPE microscopy. Avalanche photodiodes are also excellent in terms of sensitivity exhibiting quantum efficiency close to 70%–80% in the visible spectral range. Unfortunately they are high cost and the small active photosensitive area could introduce drawbacks in the detection scheme and require special de-scanning optics. CCD cameras are generally used in video rate multifocal imaging. Laser sources represent the core element for the 2PE/MPE microscope since for MPE high photon flux densities are required, > 10^24 ^photons cm^-2^s^-1^. Using radiation in the spectral range of 650–1100 nm for MPE, excitation intensities in the MW-GW cm^-2 ^have to be produced. Nowadays, laser sources suitable for 2PE can be described as "turnkey" systems, and Ti Sapphire lasers are the most utilized due to the high coincidence with the 2PE wavelengths needed for the majority of the commonly used fluorescent molecules. Other laser sources used for 2PE are Cr-LiSAF, pulse-compressed Nd-YLF in the femtosecond regime, and mode-locked Nd-YAG and picosecond Ti-Sapphire lasers in picosecond regime. Moreover the absorption coefficients of most biological samples, cells and tissues are minimised within this spectral window. Table 2 reports data on the currently available laser sources for applications in MPE microscopy and spectroscopy. The parameters that are more relevant in the selection of the laser source are average power, pulsewidth and repetition rate, and wavelength also accordingly to equation (1). The most popular features for an infrared pulsed laser are 700mW-1W average power, 80–100 MHz repetition rate, and 100–150 fs pulse width. So far, the use of short pulses and high repetition rates are mandatory to allow image acquisition in a reasonable time while using power levels that are biologically tolerable. In order to minimise pulse width dispersion problems it should be considered to operate with pulses around 150 nm. This constitutes a very good compromise both for pulse stretching and sample viability. One should always remind that a shorter pulse broadens more than a longer one. Advances in laser sources are going on considering more compact sources, large tunability range, high average power, and special designs for tailored needs at lower prices [35]. Objective lenses influence the performances of any optical microscope, and for a MPE system special considerations have to be done taking into the proper account both equations (1) and (3). New technological requisites have to be considered with respect to conventional excitation fluorescence microscope. An adequate transmission in the IR regime has to be coupled with good collection efficiency towards the ultraviolet region. Moreover, the number of components should be minimised without affecting resolution properties in order to reduce pulse widths distortions. Although the collection efficiency of the time averaged photon flux is dependent on the numerical aperture of the collecting lens, the total fluorescence generation is independent of the numerical aperture of the focusing lens when imaging thick samples. Figure 9 shows an example of optical sectioning performed through a thick sample that exploits the autofluorescence signal. This is due to the fact that the increase of intensity, obtained by a sharper focusing (high NA), is counterbalanced by the shrinking of the excitation volume. Thus the total amount of fluorescence summed over the entire space remains constant. The very relevant practical consequence of this fact is that in 2PE measurements on thick samples, assuming no aberrations, the generated fluorescence is insensitive to the size of the focal spot. As a positive consequence, a moderate variation of the laser beam size would not affect the measurements. This is a very efficient condition due to the fact that using an appropriate (non-descanned) acquisition scheme it is possible to collect all the generated fluorescence. In terms of pulse broadening, a 100 fs pulse can result between 1.14 and 1.23 times at the sample using a good lens. Once a MPE architecture has been realized, one should consider to keep under control the following parameters: power and pulse width at the sample focal plane checking for the square intensity/power behaviour, spectral separation of the emitted fluorescence including removal of the possible excitation reflections that could be particularly subtle, z-axis precise control and laser-scanning system alignment [34]. Figure 10 and figure 11 demonstrate the multiple fluorescence imaging capability and 3D multiple fluorescence imaging, respectively.

**Table 2:** MPE laser table.

**Laser Material**	**Company; Model**	**Wavelength/nm**	**Pulse Length**	**Repetition Rate**	**Power**
Ti:Sapphire	Coherent; Mira	700–980	<200 fs	76 MHz	0.7 W 1.3 W
	Spectra Physics; Tsunami	700–1000	<100 fs (or 2ps as option)	80 MHz	0.8 W 1.4 W
	Coherent; Chameleon – XR	705–980	<140 fs	90 MHz	1.7 W
	Spectra Physics; Mai Tai	710–990	120 fs	80 MHz	1.5 W
	Time Bandwidth; Pallas	780–860	<100 fs	75 MHz	500 mW
	Time Bandwidth; Tiger	780–860	<100 fs	100 MHz	400 mW
	Femtosource	750–850	<12 fs	75 MHz	400 mW 600 mW
Nd:YLF	MicroLase/Coherent Scotland; BioLite	1047	200 fs	120 MHz	500 mW
Nd:Glass	Time Bandwidth; GLX200	1058	<250 fs	100 MHz	>400 mW
Ytterbium	Amplitude Systems	1030	<200 fs	50 MHz	1 W
Cr:LiSAF	Highqlasers	850 snm	100 fs	50 MHz	>1--mW
OPO	Coherent and Spectra Physics	350–1200	100 fs		~200 mW

Commercial laser sources available for MPE microscopy and spectroscopy are reported along with main parameters. The pulse length given is the average pulse length through over the full tuning range of the system. Power is the average power at the peak of the tuning curve, typically 800 nm, or at the operating wavelength. Both Coherent Mira and Spectra Physics Tsunami systems can be pumped with a 5,8 or 10 W green laser source. When two powers are given these are for 5 W and 10 W pumped. Higher pump power may be needed for full tuning range. Between 925 and 960 nm Ti:Sapphire lasers need to be purged to ensure that no water vapour is present within the laser cavity. Computer tunable systems are sealed to prevent this complication and are endowed of a pointing stability system that reduces the need of new alignment following tuning across a wide range of wavelengths. The authors acknowledge John Girkin for providing more complete data.

## 5. Application trends and conclusions

2PE and MPE microscopes are expected to increase their impact in areas such biotechnology, neurobiology, embryology, tissue engineering, materials science where imaging can be coupled to the possibility of using the microscopes in an active way, too. Clinically, 2PE may find applications in non-invasive optical bioscopy, while in cell biology the imaging abilities are coupled to the possibility of producing localized chemical reactions. Potential applications to integrative cardiac physiology or the possibility of tracking for long time biological events in living systems point out to the ability of making direct observations of phenomena and circumstances that before could only be inferred using other approaches. The myriad of new investigation possibilities offered by 2PE/MPE microscopy enlarges so much the fields of application that it is not possible to outline in a complete way all the variations that can take place. For this reason, we give in this last paragraph asummary of main properties of MPE and a limited overview of paramount trends.

The great impact of 2PE in optical microscopy is related to the fact that it couples a three-dimensional intrinsic ability with almost five other interesting capabilities [21]. First, 2PE greatly reduces photo-interactions and allows imaging of living specimens on long time periods. Second, it allows operating in a high-sensitivity background-free acquisition scheme. Third, 2PE microscopy can penetrate turbid and thick specimens down to a depth of a few hundreds micrometers. Fourth, due to the distinct character of the multiphoton absorption spectra of many of the fluorophores 2PE allows simultaneous excitation of different fluorescent molecules reducing colocalization errors. Fifth, 2PE can prime photochemical reactions within subfemtoliter volumes inside solutions, cells and tissues.

Moreover, the advances in the field of fluorescent markers added value and potential to MPE microscopy. It is worth mentioning: the design of application suited chromophores [36]; the development and utilization of the so-called quantum dots [37]; the use of visible [38] and photoactivatable [39,40] fluorescent proteins from the green fluorescent protein (GFP) and its natural homologues to specifically engineered variants [6], the use of photoswitchable proteins to break the diffraction barrier in fluorescence microscopy at low light intensities [41].

Furthermore, this form of non-linear microscopy also supported the development and application of several investigation techniques, among them: three-photon excited fluorescence [42], second harmonic generation [43], third-harmonic generation [44], fluorescence correlation spectroscopy [45], image correlation spectroscopy [46], single molecule detection [47,48]; photodynamic therapies [49], and flow cytometry [50]. There is also an ongoing research activity to use 2PE and MPE in new fields where its special features can be advantageously applied to improve and to optimise existing schemes [51]. This covers new online detection systems like endoscopic imaging based on gradient refractive index fibres [52], the development of new substrates with higher fluorescence output [53] as well as the use of 2PE to systematically crosslink protein matrices and control the diffusion [54] and to perform localized uncaging [55]. When considering the growing interest for detection/sensing technology in medical diagnostics and biotechnology, one should not ignore the recent explosion in the use of metallic nanostructures to favourably modify the spectral properties of fluorophores and to alleviate some fluorophore photophysical constraints. Within the framework the fusion of MPE with metal-enhanced fluorescence has a powerful potential in biotechnology: from immunoassay to enhanced ratiometric sensing and DNA detection [56]. A further mention is due to biomolecular tracking in real time and in vivo. Here 2PE and MPE can be considered as the dominant technologies. One mention is for in vivo brain imaging realized by means of ea newly designed compact and portable 2PE micro endoscope recently used to visualize hippocampal blood vessels in the brains of live mice [57]. As well a first partial view into the dynamics of developmentally programmed, long-range cell migration in the mammalian thymus was obtained using in a 4D (x-y-z-t) manner 2PE. So, the movement of thymocytes was followed in real time through the cortex within intact thymic lobes [58]. All these facts point out to the consideration that MPE makes possible to perform a 7D exploration of living cells due to its inherent ability in (x-y-z-t), FLIM (Fluorescence Lifetime Imaging Microscopy), FRAP (Fluorescence Recovery After Photobleaching), FRET (Forster-fluorescence Resonance Energy Transfer) and SHG (Second Harmonic Generation) [24]. Additionally, regardless of the fact that all far field light microscopes are limited in the achievable diffraction-limited resolution, MPE is pushing modern light microscopy towards fluorescence optical nanoscopy [59,60].

**Table 3:** 2PE vs. 1PE optical microscopy.

	2PE	1PE CONFOCAL
Excitation source	Laser, IR, fs-ps pulsed, 80–100 MHz repetition rate, tunable 680–1050 nm	Laser VIS/UV CW (365, 488, 514, 543, 568, 633, 647 nm)
Excitation/emission separation	wide	close
Detectors	PMT (typical), CCD, APD	PMT (typical), CCD, APD
Volume selectivity	Intrinsic (fraction of femtoliter)	Pinhole required
Image formation	Beam scanning (or rotating disks)	Beam scanning (or rotating disks)
Deep imaging	> 500 μm (problems related to pulse shape modifications and scattering)	Approx. 200 μm (problems related to shorter wavelength scattering)
Spatial resolution	Less than confocal because of the focusing of IR radiation, compensated by the higher signal to noise ratio; pinhole increases resolution, good for high fluorescence	Diffraction limited depending on pinhole aperture
Real time imaging	Possible	Possible
Signal to noise ratio	High (especially in non descanned mode)	Good
Fluorophores	All available for conventional excitation plus new designed for 2PE	Selected fluorophores depending on laser lines in use
Photobleaching	Limited to the focal volume	Whole double cone of excitation
Contrast mechanisms	Fluorescence, high order harmonic generation, higher order n-photon excitation, autofluorescence	Fluorescence, reflection, transmission, autofluorescence.
Commercially available	Yes	Yes

Comparison of 2PE and 1PE confocal imaging systems.
